# Surgical Management of Sporadic Pancreatic Insulinomas in a Resource‐Limited Setting: A Report of Two Cases

**DOI:** 10.1155/cris/3856631

**Published:** 2026-09-08

**Authors:** Yacine Seye, Mouhamadou Laye Diop, Mohamadou Lamine Gueye, Zeinabou Lo, Mamadou Seck, Madieng Dieng

**Affiliations:** ^1^ Department of Surgery, Cheikh Anta Diop University, Dakar, Senegal, ucad.sn

**Keywords:** enucleation, hypoglycemia, insulinoma, pancreatic fistula, resource-limited setting, West Africa

## Abstract

Insulinoma is the most common functional pancreatic neuroendocrine tumor, typically benign, solitary, and sporadic, with an estimated incidence of 1–4 cases per million per year worldwide. It causes recurrent hypoglycemia through inappropriate insulin secretion, classically presenting as Whipple’s triad and confirmed by a prolonged supervised fasting test. Surgical enucleation remains the preferred treatment for small, well‐localized benign insulinomas, allowing maximal preservation of pancreatic parenchyma. We report two cases of sporadic pancreatic insulinoma managed by open enucleation at a tertiary referral center in Senegal, West Africa, where advanced diagnostic modalities such as endoscopic ultrasound (EUS), somatostatin receptor scintigraphy, and selective arterial secretagogue injection (SASI) testing were unavailable. The first case was a 30‐year‐old woman presenting with a severe hypoglycemic coma complicated by status epilepticus on postpartum day 10. Abdominal CT scan identified a pancreatic head lesion. Biochemical workup confirmed insulinoma with a fasting blood glucose of 0.30 g/L (30 mg/dL; 1.7 mmol/L), elevated fasting insulin at 32.7 µU/mL, and C‐peptide at 2.92 ng/mL. Open enucleation was performed; the postoperative course was complicated by a transient infectious syndrome managed with broad‐spectrum antibiotics. Histopathology confirmed a well‐differentiated Grade 1 neuroendocrine tumor. Complete resolution of hypoglycemic episodes was achieved with no recurrence at 1 year. The second case involved a 55‐year‐old man with a 12‐year history of recurrent neuroglycopenic episodes. CT scan revealed an isthmic pancreatic lesion. Enucleation was performed; the postoperative course was complicated by a high‐output pancreatic fistula (Grade B, ISGPS 2016 classification), managed conservatively with fasting, parenteral nutrition, and close radiological monitoring, without surgical reintervention. Histopathology confirmed a low‐grade neuroendocrine tumor. No recurrence of hypoglycemia was observed at 5 months. These cases demonstrate that open enucleation is a feasible, effective, and parenchyma‐sparing option for benign sporadic insulinomas, even in resource‐limited settings where advanced localization and intraoperative techniques remain unavailable.

## 1. Introduction

Insulinoma is the most common functional neuroendocrine tumor of the pancreas, with an estimated annual incidence of 1–4 cases per million inhabitants [1, 2]. The vast majority are benign, solitary, sporadic, and smaller than 2 cm, characteristics that largely determine the surgical strategy [2]. Clinical presentation is dominated by recurrent hypoglycemic episodes fulfilling Whipple’s triad, with biochemical confirmation provided by the 72‐h supervised fasting test demonstrating concomitant hypoglycemia, hyperinsulinemia, and elevated C‐peptide [3, 4]. Tumor localization relies on cross‐sectional imaging (CT scan, MRI) and endoscopic ultrasound (EUS), the latter offering superior sensitivity exceeding 85% [5]. CT scan sensitivity for insulinoma localization varies considerably with tumor size, ranging from less than 15% for lesions smaller than 1 cm to 60%–90% for those exceeding 1 cm, making it an imperfect but widely accessible first‐line tool [6]. When conventional imaging is inconclusive, functional techniques such as 68Ga‐DOTATATE PET/CT or selective arterial secretagogue injection (SASI) testing provide additional localization accuracy [7]. Surgical resection remains the only curative treatment, with enucleation being the preferred approach for small, benign, well‐localized tumors distant from the main pancreatic duct, given its parenchyma‐sparing properties and low risk of postoperative diabetes [8–10]. Laparoscopic enucleation is increasingly favored in high‐volume centers, though open surgery remains standard where minimally invasive expertise or equipment is unavailable [9]. The main postoperative complication is pancreatic fistula, reported in 10%–30% of cases following enucleation [11].

In sub‐Saharan Africa, the epidemiology and clinical management of insulinoma remain largely undocumented, with the available literature confined to isolated case reports and small series. This reflects both the rarity of the condition and the diagnostic challenges specific to resource‐limited settings, where advanced localization modalities, including EUS, somatostatin receptor scintigraphy, and SASI testing, are generally unavailable. In such contexts, CT scan constitutes the primary and often sole preoperative localization tool, while intraoperative adjuncts such as ultrasound and rapid hormone assays are rarely accessible. These constraints define a distinct clinical reality that warrants dedicated reporting, as published surgical experience from West Africa on this condition remains scarce.

The two cases reported herein contribute to this limited literature in several respects. Both insulinomas were localized by CT scan alone, without EUS or functional imaging; notably, the 11 mm lesion in Case 2 was successfully identified at the lower margin of conventional CT detectability [6]. Case 1 presented with hypoglycemic coma complicated by status epilepticus on postpartum day 10, a severe and clinically uncommon precipitating circumstance raising specific questions about metabolic vulnerability in the immediate postpartum period. Case 2 illustrates the diagnostic challenges of insulinoma in resource‐limited settings, with a prolonged symptomatic interval of 12 years prior to definitive diagnosis. Finally, the conservative management of a Grade B postoperative pancreatic fistula (POPF), without access to ERCP or endoscopic drainage, demonstrates that structured medical management can yield favorable outcomes even in the absence of advanced endoscopic resources. The objective of this report is to describe the surgical management of these two sporadic benign pancreatic insulinomas and to analyze the outcomes, complications, and practical implications of conservative pancreatic surgery in a resource‐limited West African setting.

## 2. Case Reports

### 2.1. Case 1

A 30‐year‐old woman with no significant medical history (G2P2) presented with a history of symptomatic hypoglycemic episodes since adolescence, relieved by sugar intake. 2 months before admission, she had developed a hypoglycemic coma on the tenth day of the postpartum period, complicated by status epilepticus, requiring hospitalization in a neurological intensive care unit and subsequent transfer to the internal medicine ward for etiological investigation.

Initial examination revealed an altered general condition (WHO performance status 2), with a capillary blood glucose of 0.66 g/L (66 mg/dL; 3.7 mmol/L), no signs of dehydration or malnutrition, a soft and depressible abdomen without palpable mass, and abdominal obesity (waist circumference 104 cm). Cutaneous findings included periumbilical purple striae and bilateral palmoplantar hyperkeratotic lesions; photographic documentation of these findings was not obtained at the time of clinical evaluation, representing an acknowledged limitation. The remainder of the clinical examination was unremarkable.

An abdominal CT scan demonstrated a hyperdense lesion at the head of the pancreas measuring 25 × 25 mm. The original digital CT images for this case were subsequently lost by the patient and could not be retrieved for inclusion in this manuscript, representing an acknowledged limitation. Biochemical workup revealed a fasting blood glucose of 0.30 g/L (30 mg/dL; 1.7 mmol/L), elevated fasting insulin at 32.7 µU/mL (reference range: 4.03–23.46 µU/mL), and C‐peptide at 2.92 ng/mL (reference range: 0.929–3.73 ng/mL), consistent with insulinoma. C‐reactive protein was elevated at 26 mg/L; remaining laboratory investigations were otherwise unremarkable.

The patient was referred to the surgical department. Intraoperative exploration identified a 3 × 3 cm tumor at the head of the pancreas without other macroscopic abnormalities. Open enucleation with drainage was performed.

The immediate postoperative course was complicated on day 2 by an infectious syndrome with leukocytosis at 15,570/µL and CRP at 102.8 mg/L. Broad‐spectrum antibiotic therapy was initiated with favorable clinical and biological evolution. On day 7, the abdomen was soft, the wound clean, and the drain nonproductive; it was therefore removed. The patient was discharged on day 8 with normalized blood glucose and referred for endocrinological and neurological follow‐up.

Histopathological examination confirmed a well‐differentiated Grade 1 neuroendocrine tumor.

At 1 month postoperatively, the patient had no further hypoglycemic episodes and demonstrated a favorable clinical evolution with complete wound healing. At 3 months and at 1 year of follow‐up, there was no recurrence of symptoms, the overall general condition was good, fasting blood glucose was normalized at 1.02 g/L (102 mg/dL; 5.7 mmol/L), and the patient maintained regular endocrinological and neurological follow‐up. She continued anticonvulsant therapy with carboxamide derivatives.

### 2.2. Case 2

A 55‐year‐old man with no known medical or surgical history, an active smoker (10 pack‐years), presented with a 12‐year history of recurrent episodes characterized by vertigo, intense hunger followed by confusion, diplopia, psychomotor agitation, and loss of consciousness, occurring during fasting or after prolonged physical effort. Episodes were rapidly relieved by oral sugar intake. Due to their frequency and functional impact, the patient consulted internal medicine.

Clinical examination was normal except for a moderate autonomic syndrome. The patient was overweight (BMI 29.2 kg/m^2^) with a capillary blood glucose of 0.319 g/L (31.9 mg/dL; 1.8 mmol/L).

**Figure 1:**
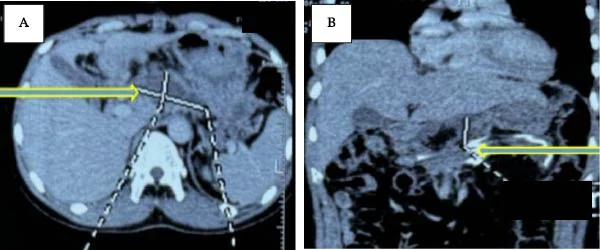
Preoperative abdominal CT scan findings in Case 2. (A) Axial arterial‐phase CT image demonstrating a well‐defined hypervascular lesion (arrow) measuring 11 × 11 mm at the pancreatic isthmus, with characteristic enhancement consistent with a neuroendocrine tumor. (B) Coronal reconstruction confirming the isthmic location of the lesion (arrow) in relation to the adjacent pancreatic parenchyma and peripancreatic vessels. All patient‐identifying information has been removed.

A supervised fasting test confirmed deep hypoglycemia (mean blood glucose 0.3 g/L; 30 mg/dL; 1.7 mmol/L) with elevated fasting insulin at 31.67 µU/mL (reference range: 2.6–25 µU/mL) and C‐peptide at 2.42 µg/L (reference range: 0.8–4.2 µg/L), consistent with insulinoma. Abdominal CT scan demonstrated a pancreatic isthmic lesion measuring 11 × 11 mm (Figure 1).

**Figure 2:**
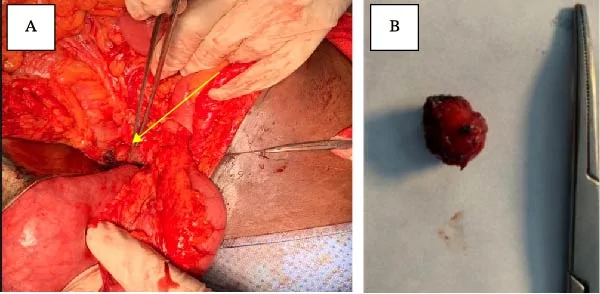
Intraoperative findings in Case 2. (A) Intraoperative photograph demonstrating the insulinoma (arrow) at the pancreatic isthmus prior to enucleation; the tumor measured approximately 1.5 cm with a regular surface and well‐defined margins. (B) Resected operative specimen following enucleation, showing the intact tumor with surrounding pancreatic tissue preserved.

Exploratory laparotomy was performed. Intraoperative exploration identified a tumor with a regular surface measuring 1.5 cm at the pancreatic isthmus. Open enucleation was performed, followed by drainage (Figure 2).

**Figure 3:**
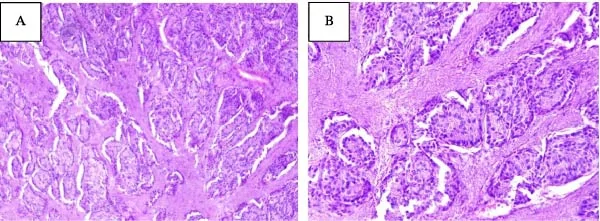
Histopathological examination of the resected insulinoma in Case 2, consistent with a low‐grade (Grade 1) well‐differentiated neuroendocrine tumor. (A) Hematoxylin‐eosin staining at × 100 magnification showing a well‐circumscribed tumor composed of uniform neoplastic cells arranged in nests and trabeculae with scant mitotic activity. (B) Hematoxylin‐eosin staining at × 200 magnification demonstrating neoplastic cells with round‐to‐oval nuclei, stippled chromatin, and abundant cytoplasm, characteristic of a neuroendocrine neoplasm.

Histopathological examination was consistent with a low‐grade neuroendocrine tumor (Figure 3).

The immediate postoperative course was complicated on day 7 by a high‐output pancreatic fistula (1 L/24h). The patient remained hemodynamically stable. A control abdominal CT scan revealed a 91 mL retroperitoneal collection communicating with the drain. Drain fluid amylase exceeded four times the upper limit of normal, confirming the diagnosis. Conservative management was initiated, including bowel rest, total parenteral nutrition, and close clinical and radiological surveillance.

On day 14, the fistula persisted with an output of 300 mL/24h. The patient was transfused due to moderate anemia and a decreased prothrombin time (62%, INR 1.4). On day 21, the fistula output had reduced to 150 mL/24h. Abdominal ultrasound showed moderate peritoneal effusion. General condition remained stable and the surgical wound had healed. With the normalization of blood glucose, the patient was discharged for outpatient follow‐up.

**Figure 4:**
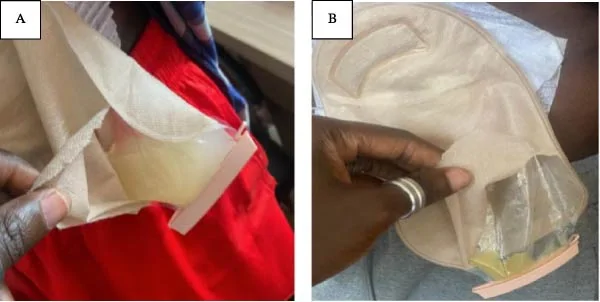
Postoperative course of the Grade B pancreatic fistula in Case 2. (A) Photograph at postoperative month 1 showing the drainage system in situ with persistent but low‐output, directed fistula, with the collection bag demonstrating reduced secretion compared to the immediate postoperative period. (B) Photograph at postoperative month 2 showing further reduction in fistula output (~30 mL/24h), consistent with progressive spontaneous resolution under conservative outpatient management.

At 1 month, the fistula was low‐output and well‐directed, with continuation of outpatient surveillance. At 2 months, the output was estimated at 30 mL/24h and was tapering (Figure 4). The patient had lost significant weight (86.4 kg compared to 122 kg preoperatively) and had no further hypoglycemic episodes.

At 5 months postoperatively, the patient continues regular follow‐up. The drainage system is maintained for surveillance of the residual fistula, which is evolving favorably.

## 3. Discussion

### 3.1. Epidemiology

In sub‐Saharan Africa, robust epidemiological data are virtually absent, and the condition is likely underdiagnosed due to limited access to biochemical workup and specialized imaging. To the best of the authors’ knowledge, the existing literature from this region is confined to isolated case reports and small series, reflecting both the rarity of the condition and the significant diagnostic barriers inherent to resource‐limited healthcare settings. The prolonged symptomatic interval of 12 years in Case 2 before definitive diagnosis illustrates these challenges and underscores the need for greater awareness of insulinoma among primary care and emergency providers in this context.

### 3.2. Clinical Presentation

Case 1 is noteworthy for its severe and uncommon precipitating circumstance, hypoglycemic coma complicated by status epilepticus on postpartum day 10. Although the patient had experienced episodic hypoglycemia since adolescence, suggesting a longstanding subclinical disease, the postpartum period appears to have precipitated acute decompensation. This phenomenon is well recognized but rare: only ~25 cases of insulinoma during the perinatal period had been reported in the English literature as of 2021, with ~35% diagnosed immediately postpartum [12, 13]. The underlying mechanism relates to the protective role of pregnancy‐induced insulin resistance, which attenuates the clinical expression of hyperinsulinism during gestation. Following delivery, the abrupt resolution of this resistance unmasks the tumor’s secretory activity, precipitating severe hypoglycemia [12, 13]. The additional metabolic demands of the immediate postpartum period, including lactation, relative caloric restriction, and physiological stress, may further lower the hypoglycemic threshold in susceptible patients, though this remains a pathophysiological inference based on general endocrine principles rather than insulinoma‐specific data [12]. Clinicians should therefore maintain a high index of suspicion for insulin‐secreting tumors in postpartum women presenting with unexplained severe hypoglycemia, particularly those with a history of episodic symptoms predating pregnancy.

### 3.3. Biochemical Diagnosis

The biochemical diagnosis was established in both cases through the demonstration of endogenous hyperinsulinemic hypoglycemia during a supervised fasting test, consistent with international diagnostic criteria [4]. Fasting blood glucose was markedly low in both patients; 0.30 g/L (30 mg/dL; 1.7 mmol/L) in Case 1 and 0.319 g/L (31.9 mg/dL; 1.8 mmol/L) in Case 2, with concomitantly elevated fasting insulin levels of 32.7 µU/mL (reference range: 4.03–23.46 µU/mL) in Case 1 and 31.67 µU/mL (reference range: 2.6–25 µU/mL) in Case 2. C‐peptide values were within the upper normal range in both cases, effectively excluding exogenous insulin administration as a cause [4]. Proinsulin levels were not measured, representing a minor limitation; however, the biochemical profile in both cases was sufficiently characteristic to confirm the diagnosis without this additional assay.

Approximately 10% of insulinomas occur in the context of Multiple Endocrine Neoplasia type 1 (MEN1), which is typically associated with multiple pancreatic neuroendocrine tumors and carries distinct surgical and surveillance implications [2, 3, 14]. In both cases presented, the tumors were solitary on imaging and the clinical presentation was consistent with sporadic disease.

In both cases, a targeted biochemical workup was performed to exclude MEN1. Serum calcium levels were normal in Case 1 (2.39 mmol/L; reference range: 2.20–2.60 mmol/L) and Case 2 (2.36 mmol/L; reference range: 2.20–2.60 mmol/L). Intact PTH was within normal limits in both patients (Case 1: 36 pg/mL; Case 2: 53 pg/mL; reference range: 10–65 pg/mL). Prolactin levels were normal in both cases (Case 1: 11 ng/mL, reference range: 5–25 ng/mL; Case 2: 11 ng/mL, reference range: 3–15 ng/mL). The absence of biochemical evidence of hyperparathyroidism or hyperprolactinemia, combined with the solitary nature of the pancreatic lesions on imaging and the absence of personal or family history of endocrine neoplasia, effectively excluded MEN1 in both patients. Formal genetic testing for MEN1 germline mutation was not performed, representing a recognized limitation in a resource‐limited setting where such testing is unavailable.

### 3.4. Tumor Localization

Preoperative localization was achieved by CT scan alone in both cases, without recourse to EUS, somatostatin receptor scintigraphy, or SASI testing, none of which are available at our institution. Furthermore, our institution does not have a picture archiving and communication system (PACS), precluding direct digital export of CT images—a recognized technical limitation in resource‐limited settings. CT scan sensitivity for insulinoma localization varies considerably with tumor size, ranging from less than 15% for lesions smaller than 1 cm to 60%–90% for those exceeding 1 cm [6]. In Case 1, the 25 mm cephalic lesion was well within the range of reliable CT detection. In Case 2, the 11 mm isthmic lesion was successfully identified at the lower margin of conventional CT detectability, an outcome that may not be reproducible for smaller occult lesions.

In settings where CT fails to localize the tumor, the SASI test involving selective intra‐arterial calcium injection into the major pancreatic arteries with hepatic venous insulin sampling represents an important adjunct for tumor regionalization, with a reported sensitivity of 93% in a dedicated insulinoma cohort [15]. This technique is currently unavailable at our institution, as in most West African referral centers. Had CT failed to localize either tumor, surgical exploration with intraoperative bimanual palpation of the pancreas would have remained the only available fallback, correctly identifying insulinoma in 83% of cases in one series [15], with success rates highly dependent on tumor size and surgical expertise [9, 10]. This underscores the critical role of surgical skill in resource‐limited settings, where technical adjuncts cannot substitute for thorough and systematic manual exploration of the pancreas.

### 3.5. Surgical Management and Intraoperative Considerations

Open enucleation was performed in both cases, consistent with established guidelines recommending this parenchyma‐sparing approach for small, benign, well‐localized insulinomas distant from the main pancreatic duct [9, 10]. Although laparoscopic enucleation is increasingly favored in high‐volume centers for its reduced morbidity and shorter hospital stay [9], it was not considered in the present cases due to the absence of appropriate laparoscopic equipment and expertise, a constraint common to many surgical centers across sub‐Saharan Africa. The favorable outcomes achieved through open enucleation confirm that this approach remains a valid and effective alternative when minimally invasive surgery is unavailable [16].

Intraoperative biochemical confirmation of complete tumor resection, through serial measurement of blood glucose and insulin levels before and after enucleation, was not performed in either case. This represents a recognized limitation of our intraoperative management. Rapid normalization of blood glucose following tumor removal may provide real‐time evidence of complete excision of the insulin‐secreting lesion, though glycemic rebound is not universally observed and should not be the sole criterion for assessing resection completeness [17, 18]. In resource‐limited settings where laboratory‐based intraoperative assays are unavailable, point‐of‐care capillary glucose monitoring represents a practical, low‐cost, and readily accessible alternative that the authors consider worthy of systematic incorporation into the intraoperative protocol for insulinoma surgery. In the present cases, clinical resolution of hypoglycemia during the immediate postoperative period provided indirect evidence of complete resection, subsequently confirmed by histopathology.

### 3.6. Postoperative Complications

POPF is the most frequent complication following enucleation, with reported incidence rates of 10%–30% depending on tumor proximity to the main pancreatic duct and the degree of parenchymal manipulation [9]. Case 2 developed a high‐output POPF on postoperative day 7, classified as Grade B according to the 2016 ISGPS definition, as it required prolonged drain maintenance beyond 3 weeks and modification of the postoperative management plan without necessitating surgical reintervention [19]. A CT scan confirmed a 91 mL retroperitoneal collection communicating with the drain, and drain fluid amylase exceeded four times the upper limit of normal, satisfying the biochemical diagnostic criterion [19].

Management followed a conservative multidisciplinary approach comprising bowel rest, total parenteral nutrition, and close clinical and radiological surveillance. Fistula output decreased progressively from 1 L/24h at onset to 300 mL/24h at day 14, 150 mL/24h at day 21, and 30 mL/24h by postoperative month 2, with a favorable trajectory at month 5. The patient required blood transfusion for moderate anemia with decreased prothrombin time (62%, INR 1.4) at day 14, reflecting the systemic impact of a prolonged high‐output fistula.

ERCP with pancreatic duct stenting was considered as a therapeutic option, as transpapillary drainage can reduce fistula output by decompressing the main pancreatic duct [19]. However, ERCP was unavailable at our institution, and patient transfer was precluded by clinical and logistical constraints. The progressive resolution achieved through conservative management alone, without endoscopic or surgical reintervention, demonstrates that a structured nonoperative approach remains a viable first‐line strategy for Grade B POPF in resource‐limited settings, provided that rigorous multidisciplinary monitoring is maintained. This case also highlights the considerable financial burden imposed by prolonged hospitalization and parenteral nutrition on patients and families in contexts where healthcare expenditure is largely out‐of‐pocket, reinforcing the broader imperative for healthcare system reforms aimed at improving access to affordable surgical care in West Africa.

### 3.7. Histopathology

Histopathological examination confirmed well‐differentiated Grade 1 neuroendocrine tumors in both cases, consistent with the predominantly benign behavior of sporadic insulinomas [1, 2]. For Case 2, hematoxylin‐eosin stained sections are provided (Figure 3). For Case 1, histopathological analysis was performed by an external pathology laboratory; the anatomopathological report confirming a well‐differentiated Grade 1 neuroendocrine tumor is available and can be provided to the editorial office upon request. Despite our best efforts, the original histological slides and digital images could not be retrieved for inclusion in this manuscript, as the external laboratory was unable to locate the archived material at the time of manuscript preparation. This represents an acknowledged limitation of the present report.

Immunohistochemical staining, including synaptophysin, chromogranin A, Ki‐67/MIB‐1 index, and insulin, was not performed in either case, as these techniques were unavailable at our institution’s pathology department at the time of diagnosis. This represents a recognized limitation of our histopathological workup. The diagnosis of well‐differentiated Grade 1 neuroendocrine tumor was established on morphological grounds alone, based on hematoxylin‐eosin staining demonstrating characteristic neuroendocrine architecture with uniform cells, stippled chromatin, and low mitotic activity. While immunohistochemical confirmation is the international standard for neuroendocrine tumor diagnosis and grading [14], the absence of these stains is an acknowledged constraint in resource‐limited settings and does not preclude the histomorphological diagnosis in the context of a consistent clinical and biochemical presentation.

### 3.8. Prognosis and Follow‐up

The prognosis of benign insulinoma following complete surgical resection is excellent, with cure rates consistently reported above 90% and low recurrence rates in long‐term follow‐up series [20]. Both patients demonstrated complete clinical resolution of hypoglycemic episodes following enucleation. Case 1 remained free of recurrence at 1 year, with a normalized fasting blood glucose of 1.02 g/L (102 mg/dL; 5.7 mmol/L), continuing anticonvulsant therapy for residual epilepsy attributable to the prior status epilepticus. Case 2 showed no hypoglycemic recurrence at 5 months, with ongoing radiological surveillance of the residual pancreatic fistula. Follow‐up in both cases was conducted through a multidisciplinary approach involving surgery, endocrinology, and neurology, which we consider essential for optimizing long‐term outcomes and detecting potential recurrence.

These two cases demonstrate the feasibility and effectiveness of open enucleation for sporadic benign pancreatic insulinomas in a resource‐limited West African setting. Successful outcomes were achieved through CT–based preoperative localization, careful open surgical technique, and structured postoperative multidisciplinary management, without access to EUS, functional imaging, SASI testing, intraoperative hormone assays, or endoscopic fistula treatment. While these limitations are real and should not be minimized, they define a practical management framework within which function‐preserving pancreatic surgery can be safely performed. Rigorous patient selection, surgical expertise, and early recognition and conservative management of complications remain the cornerstones of optimal outcomes in this context. These observations contribute to the scarce published literature on insulinoma management in sub‐Saharan Africa and may serve as a practical reference for surgeons facing similar resource constraints across the continent.

## Author Contributions

**Yacine Seye**, **Mouhamadou Laye Diop**, and **Mohamadou Lamine Gueye**: conceptualization, methodology, supervision. **Yacine Seye**, **Mouhamadou Laye Diop**, and **Mamadou Seck**: investigation (surgical procedures). **Mouhamadou Laye Diop** and **Yacine Seye**: writing – original draft. **Zeinabou Lo**, **Mamadou Seck**, and **Madieng Dieng:** data curation, writing – review and editing.

## Funding

This research received no specific grant from any funding agency in the public, commercial, or not‐for‐profit sectors.

## Disclosure

All authors have read and approved the final version of the manuscript.

## Ethics Statement

This study was conducted in accordance with the ethical principles outlined in the Declaration of Helsinki. As a retrospective case report involving two patients with de‐identified clinical data, formal ethical committee approval was not required per institutional policy. Both patients provided written informed consent for the publication of their clinical data and images.

## Consent

Written informed consent was obtained from both patients for the publication of this case report and any accompanying images. Copies of the written consent forms are available for review by the Editor‐in‐Chief of this journal upon request. All patient‐identifying information, including names visible on imaging studies, has been removed or obscured prior to submission.

## Conflicts of Interest

The authors declare no conflict of interest.

## Data Availability

The data that support the findings of this study are available from the corresponding author upon reasonable request.
